# Teachers’ data literacy for learning analytics: a central predictor for digital data use in upper secondary schools

**DOI:** 10.1007/s10639-023-11772-y

**Published:** 2023-04-11

**Authors:** Konstantinos Michos, Maria-Luisa Schmitz, Dominik Petko

**Affiliations:** Institute of Education, University of Zurich, Kantonsschulstrasse 3, Zurich, 8001 Switzerland

**Keywords:** Digital data, Teachers, Schools, Data literacy, Learning analytics

## Abstract

Since schools increasingly use digital platforms that provide educational data in digital formats, teacher data use, and data literacy have become a focus of educational research. One main challenge is whether teachers use digital data for pedagogical purposes, such as informing their teaching. We conducted a survey study with N = 1059 teachers in upper secondary schools in Switzerland to investigate teacher digital data use and related factors such as the available technologies in schools. Descriptive analysis of the survey responses indicated that although more than half of Swiss upper-secondary teachers agreed with having data technologies at their disposal, only one-third showed a clear tendency to use these technologies, and only one-quarter felt positively confident in improving teaching in this way. An in-depth multilevel modeling showed that teachers’ use of digital data could be predicted by differences between schools, teachers’ positive beliefs towards digital technologies (will), self-assessed data literacy (skill), and access to data technologies (tool) as well as by general factors such as frequency of using digital devices in lessons by students. Teacher characteristics, such as age and teaching experience, were minor predictors. These results show that the provision of data technologies needs to be supplemented with efforts to strengthen teacher data literacy and use in schools.

## Introduction

The use of educational data to inform pedagogical decisions has been explored in research related to schools. In this context, data literacy for teaching (Mandinach & Gummer, 2016) or pedagogical data literacy (Mandinach, 2012) is an important competence that affects teachers’ data-informed classroom practices and contributes to better student learning (Dunn et al., 2013; Kippers et al., 2018; Poortman & Schildkamp, 2016). According to a recent review, more research is needed on data literacy to integrate a situated view of educational data into everyday life with an emphasis on context-specific evaluations (Gebre, 2022). Even before the digital era, school teachers had access to a variety of educational data to inform their pedagogical decisions at the classroom level (e.g., student achievement data, student attendance data, and student products). Currently, the increasing use of digital learning environments and student information systems in schools enables the storing and analysis of additional types of educational data, and digital formats offer further opportunities to store, process, and display data for pedagogical decision making.

Students interact daily with learning management systems, digital learning applications, or digital communication platforms in their everyday practice, and this became even more pervasive during and after the COVID-19 pandemic. The analysis and use of digital educational data are of high relevance in educational research, and emerging fields, such as learning analytics and educational data mining, can enrich traditional approaches to understanding and optimizing the conditions for teaching and learning (Papamitsiou & Economides, 2014). However, new forms of collecting, analyzing, and using digital data in schools with digital platforms have just started to be explored (Kovanovic et al., 2021; Mandinach & Abrams, 2022; Sousa et al., 2021). Although learning analytics tools are increasingly available in school contexts (Kovanovic et al., 2021), research related to teachers’ uses of digital data is still in its infancy.

Previous research on teacher data literacy in schools has explored relevant factors that affect teachers’ data use (Mandinach & Gummer, 2016; Mandinach & Jimerson, 2016). The present study seeks to further investigate different factors that affect teachers’ digital data use across different digital platforms in schools, with reference to technology integration research. We conducted a survey study in upper secondary schools in Switzerland with the aim of enhancing the understanding of digital, data-informed teacher practices in schools and discussing the consequences of the future use of learning analytics in K–12 contexts.

## Teacher data use in schools and digital transformation

The successful implementation of data-driven instruction and learning analytics tools in classrooms also depends on teacher practices. In a literature review, Datnow and Hubbard (2016) identified several factors affecting teachers’ effective use of student data, such as teachers’ beliefs, efficacy, and capacity to work with educational data (Bertrand & Marsh, 2015). Other studies have focused on the various forms of support given to teachers to use data effectively. For instance, access to technologies that provide data or relevant computer data systems, the available, structured time to reflect with data, and school leadership have been shown to influence teacher data use in schools (Rangel et al., 2016). A relatively high number of studies have focused on teacher data literacy with the aim of studying teachers’ engagement and the pedagogical uses of student data (Henderson & Corry, 2020). Mandinach & Gummer (2016) described teacher data literacy as “*the ability to transform information into actionable instructional knowledge and practices by collecting, analyzing, and interpreting all types of data (assessment, school climate, behavioral, snapshot, etc.) to help determine instructional steps*” (p. 367). This involves various factors, such as teacher beliefs, teacher pedagogical content knowledge, and experiences with data inquiry. Most studies on teacher data literacy conclude that a better framework for capacity building and relevant teacher training (Farley-Ripple & Buttram, 2015) is required to improve teacher data use.

The current digital transformation of schools has changed practices regarding the collection, management, analysis, and processing of educational data. According to Jarke and Breiter (2019) digital data

*“allow for the analysis of different educational practices to a degree of complexity not previously possible and to a much greater extent, as they can be very detailed, cover a more complete scope and can be flexibly combined. This is increasingly happening in real time due to the power of computers and algorithms. In the near future, sensors will provide further data. As such digital data not only serve to support decisions, but also fundamentally change the organisation of learning and teaching”* (p.3).

Compared to findings in previous research on teacher data literacy, the use of digital technologies in schools now enables the automatic collection and production of “digital trace data” (Breiter & Hepp, 2018) in real time. Teachers are considered the main educational stakeholders who are presented with these new forms of data, and this happens through access to data visualization tools, teacher dashboards, data technologies, and student information systems (Sampson et al., 2022). The rise of digital trace data and data technologies in education is expected to influence research and practices in learning analytics (Papamitsiou & Economides, 2014) in the school context (Kovanovic et al., 2021; Sousa et al., 2021). However, although empirical evidence on teachers’ use of traditional educational data has shown positive effects on student learning, there is a lack of evidence regarding the use of these new possibilities in using digital student data in schools.

## Teachers’ use of digital data and learning analytics in schools

The use of digital platforms that provide data displays to present real-time or more detailed insights into learning processes by using sophisticated statistical models (e.g., artificial intelligence techniques) to adapt to student needs and to provide personalized learning experiences leads to new skill demands for teachers (Celik et al., 2022; Howard et al., 2022; Mandinach & Abrams, 2022; Sousa et al., 2021; Sampson et al., 2022). Although teachers have more opportunities to use these new data technologies, there is still no systematic research on how these technologies are used effectively in practice. In particular, there is a lack of large-scale studies that seek to understand whether and why teachers engage with these data technologies in schools.

Some studies have explored teachers’ perceptions or use of digital data in school contexts on a small scale. For instance, Jover (2019) conducted interviews in Australian schools and discovered both positive and negative perceptions of teachers with regard to digital student data. Among the positive perceptions were that teachers are eager to work with digital data because they can adapt their teaching strategies to their students. They also felt satisfied when they saw visual forms of data and perceived the availability of data on digital platforms as easy. Among the negative teachers’ perceptions were that engagement with digital data could be a time-consuming process, the risk of overestimating quantitative data, and possible overload due to data deluge. This is in line with other empirical studies showing that teachers’ beliefs and previous experiences are related to teachers’ sense-making of data (Bertrand & Marsh, 2015).

Large-scale survey studies have evaluated analog data for teachers or assessment data without focusing on data collected on digital learning platforms. These studies have shown that teachers’ intentions to use data, teachers’ beliefs, and student outcomes are related to teacher data use (Keuning et al., 2019; Kippers et al., 2018; Pierce et al., 2013; Prenger & Schildkamp, 2018; Yan et al., 2021). Relevant theoretical frameworks were used to explain factors influencing teachers data use in schools including the theory of planned behaviour (TPB) (Ajzen, 1991) and technology acceptance model (TAM) (Davis, 1989). Prenger & Schildkamp (2018) used the TPB and showed that perceived control; the degree to which teachers have autonomy to make instructional changes based on data, their beliefs that data can lead to improvement instead of their experiences and their intentions to use data significantly predicted data use in schools. Schildkamp (2019) further explains that big data have just started to be explored for school improvement and teacher use but socio-technical and ethical challenges should be considered in school contexts. Mavroudi et al. (2021) conducted a survey study based on TAM to understand school teachers’ views about learning analytics. Explorative analysis showed that perceived usefulness of learning analytics applications was positive but teachers had moderate views with respect to their scepticism, willingness, and readiness about learning analytics in their school practice. The survey study by Hase et al. (2022) made the first attempt to identify factors that support the integration of digital learning platforms and learning analytics in teaching at a larger scale. The authors applied the TPB to demonstrate that teacher competencies, operationalized as perceived behavior control, relate to the use of learning analytics. However, although teacher competencies with data seem to influence the use of digital data, there is still a gap in research on identifying related factors that affect teacher use of learning analytics collected on digital platforms in K–12 on a larger scale (Sousa et al., 2021).

Given the current state of research, the available evidence suggests the need for a better investigation of teacher characteristics, such as their age, technological skills, and gender, and teacher factors, such as data literacy, professional routines, and pedagogical knowledge (Hase et al., 2022; Ifenthaler, 2021; Kovanovic et al., 2021; Leeuwen et al., 2019, 2021; Molenaar & Knoop-van Campen, 2018; Sousa et al., 2021). In addition, further investigation of the sociotechnical system and the school context that shapes teachers’ practices with regard to data use (e.g., their school environment and technological infrastructure) is needed (Datnow & Hubbard, 2016; Michos et al., 2018; Knauder & Koschmieder 2019; Sousa et al., 2021). As there are few dedicated studies on these contextual factors influencing the uptake of learning analytics in schools, it can be assumed that similar factors, such as those in general technology integration research, might play a central role. Beyond the availability of suitable hardware and software, teacher skills and teacher beliefs have been identified as core factors of technology integration in education (Davies & West, 2014; Niederhauser & Lindstrom, 2018; Tondeur et al., 2020). Therefore, research on learning analytics technologies in schools could be guided by models such as the “Will-Skill-Tool” model (WST model), which explains a large portion of the variance in the pedagogical uptake of technologies by teachers (Knezek et al., 2000; Petko, 2012; Petko & Prasse, 2018)

### Will-skill-tool model with regard to learning analytics in schools

The WST model focuses on the proficiency of teachers (Christensen & Knezek, 2016) and postulates that the teachers` beliefs with regard to digital technologies (will), their technical skills (skill), and the digital equipment available for the teacher (tool) are the most important predictors for technology integration (Knezek et al., 2003; Niederhauser & Lindstrom, 2018). This postulation has been confirmed by many studies showing that those three predictors play a very important role in technology integration (Agyei & Voogt, 2011; Petko, 2012; Farjon et al., 2019; Knezek et al., 2003; Knezek et al., 2000; Knezek & Christensen, 2008, 2016; Morales Velazquez, 2007; Pozas & Letzel, 2021; Sasota et al., 2021; Sawyerr & Agyei, 2022). In the majority of older studies technology integration was measured as the teachers` familiarity, beliefs, and self-efficacy with regard to digital technologies that can be confounded with the will- and skill-component of the WST model (see for example Agyei & Voogt 2011; Knezek et al., 2003; Knezek & Christensen, 2016). To avoid this confounding problem other studies have operationalized technology integration as the frequency of technology use by teachers and students in class and have investigated it in combination with key components of the WST model (see Petko, 2012; Schmitz et al., 2022; Sasota et al., 2021;). However, in a European large scale assessment study it was found that will-, skill- and tool-components were only of minor importance for the frequency of technology use (e.g., computer and internet applications) by students and teachers in class (Schmitz et al., 2022). Instead, Schmitz et al. (2022) proposed to examine differential effects of the WST model components on other digital practices of teachers.

Although the above studies focused on digital technologies, there is no study with respect to digital data use by teachers. The WST components align with previous studies reaviling that teachers` beliefs (will) and their data literacy (skill) are related to teachers data use (Hase et al., 2022; Ifenthaler, 2021; Keuning et al., 2019; Kippers et al., 2018; Kovanovic et al., 2021; Leeuwen et al., 2019, 2021; Molenaar & Knoop-van Campen, 2018; Pierce et al., 2013; Prenger & Schildkamp, 2018; Sousa et al., 2021; Yan et al., 2021). Moreover, several studies stressed the need of analyzing the technological infrastructure in schools and the availability of data technologies (tool) as an additional predictor for teachers` data use (Datnow & Hubbard, 2016; Michos et al., 2018; Sousa et al., 2021).Thus, in this study, we focus on the use of digital data by school teachers and investigate related teacher factors based on typical factors that are considered in general technology integration research in schools, in particular the will-, skill- and tool-components of the WST model. Based on those theoretical considerations, we investigated the following research questions:

1. What is the availability of different digital platforms with learning analytics in schools and the levels of self-reported teacher data use and data literacy?
2. Which factors predict digital data use for teaching?
3. To what extent do digital data use and data literacy differ according to the types of digital platforms used in their schools?

With the first research question we aim to analyze teacher-related factors about the use of digital data in schools at a large scale. Regarding the second research question, we assume that teachers’ positive beliefs toward digital technologies (will) and their data literacy (skill) as well as their access to data analytics platforms (tool) and their general use of these technologies are positively related to their use of digital data when taking variance within schools into account (Cho & Wayman, 2014; Sousa et al., 2021). Regarding the third research question, we assume that teachers’ use of digital data and data literacy will differ significantly according to the types of digital platforms used in their schools.

## Research methods

### Context and sample

A survey study was conducted to answer the research questions. A total of 1059 in-service teachers from 54 schools in a German-speaking canton of Switzerland participated in this study. All teachers who teach in the second grade of upper secondary education received an invitation by e-mail to respond to an online questionnaire. As the invitations were distributed by the school headmasters, a precise response rate was difficult to estimate. Overall, 17.5% of upper secondary education teachers in the canton responded to the survey, representing 74% of the schools. Data collection took place between September and November 2021. Overall, 50% of the responders were female, 47.3% were male, and 2.6% were identified as diverse. Their average age was 46.36 years old (*SD* = 9.87), and their average teaching experience was 15.56 years (*SD* = 9.63), with a range of 42 years. Of the responders, 53.2% were teaching in general baccalaureate schools (so-called “Gymnasiums”) and technical baccalaureate schools (Fachmittelschule), and 46.8% were teaching in vocational schools.

### Instruments

Based on our aim to integrate the WST model in the use of digital data by teachers, we initially evaluated positive beliefs toward digital technologies in general (will). We used a scale (Cronbach’s alpha = 0.889) by Petko (2012) that included four items: “Through the use of digital technologies, I can improve the quality of my teaching,” “The performance of learners can be increased when using digital technologies,” “When learners work with digital technologies, they can improve learning and their working strategies,” and “Through the use of digital technologies, the work efficiency of learners can be increased.” The four items used a 5-point Likert scale that ranged from “Strongly disagree” to “Strongly agree.”.

To measure the use of digital data by teachers and related factors, we adopted Wayman et al.’s (2016) instrument. This instrument evaluates teachers’ pedagogical actions based on data with the purpose of improving instruction in school contexts. We used an item related to teachers’ data literacy regarding the use of digital student data (skill): “I know how to improve my teaching and student learning by analyzing digital student data.” Another item evaluated whether teachers have access to available technologies for the analysis of digital student data in their schools (tool): “I have adequate technology to examine digital student data.” Finally, another item evaluated the pedagogical use of digital student data (data use): “I use digital student data to plan and adjust my teaching.”. The three items included a 5-point Likert scale ranging from “Strongly disagree” to “Strongly agree.”.

We evaluated additional factors, in particular, the frequency of using digital devices in lessons (Barras & Petko, 2007) with two items, one concerning the teacher: “How often do you use a digital device in your lessons?”, and one concerning the students: “How often do students work with a digital device in your lessons?”. The two items included a 6-Point Likert Scale (1-Never, 2-Less than once a month, 3-Once a week to once a month, 4-Several times a week, 5-Almost every day, 6-Almost in every lesson). Lastly, we used three items regarding teachers’ characteristics about their years of teaching experience, gender, and age.

#### Data technologies in schools

An online questionnaire was administered to school principals to examine digital transformation practices. From this questionnaire, we used one open question that asked school principals which digital platforms were officially used in their schools. The study comprised answers from *N* = 105 school principals from *N* = 53 schools.

### Data analysis

We used descriptive statistics to report teachers’ responses on digital data use for teaching with the three items related to data use (availability of data technologies, digital data use by teachers, and teachers’ data literacy) and all related factors (teachers’ positive beliefs, digital device use by teachers, digital device use by students, age, teaching experience, and gender). We also combined the data regarding the different digital platforms from the school principals’ questionnaires to associate teachers with the digital platforms in their schools. We initially reviewed all responses regarding the different platforms for each school and categorized them according to their main functionality as follows: “Learning Management System (LMS)” such as Moodle, “Apps for learning (Apps)” such as Kahoot, “Reading-Writing” such as Adobe Reader or Word Office, “Videoconference” such as Zoom, “Cloud-based collaboration” such as OneDrive, every cloud-based platform for sharing documents, “LMS/Videoconference/Collaboration” such as Microsoft Teams, and “Other” such as school administration software. We also categorized the platforms according to their functionality to provide data analytics as follows: “Digital platforms with analytics” and “Digital platforms without analytics.” All platforms with analytics functionalities provide teachers with an overview of student activity with dashboards or control panels. Although these platforms were officially used in the schools by considering the schools’ technological infrastructure, additional platforms might be used out of the school context (e.g., platforms selected by students for studying in their home or teachers to prepare for their teaching). However, we did not consider these platforms in order to understand how the schools’ technological infrastructure might have affected the use of digital data by teachers.

Since our data included teachers who were nested in schools, a multilevel linear model with two levels was used to investigate the factors influencing teachers’ digital data use. After checking the statistical prerequisites, we used a multilevel model with random intercept and centered the predictors based on group means (Enders & Tofighi, 2007; Field, 2013; Peugh, 2010). The dependent variable was the digital data use by teachers. The predictor variables were positive beliefs toward technology use (will), teachers’ data literacy (skill), availability of data technologies (tool), teachers’ characteristics (gender, age, teaching years), and frequency of using digital devices by teachers and students. The variable of digital device use by teachers was recoded as 1 for the first five Likert scale responses (1-Never, 2-Less than once a month, 3-Once a week to once a month, 4-Several times a week, 5-Almost every day) and 2 for the sixth response (6-Almost in every lesson) in order to avoid a ceiling effect since participants’ responses clustered toward the high end (highest value). We initially estimated an unconditional model (null model) to understand whether there were differences in teachers’ digital data use for teaching at the teacher and school levels. We then created a model based on the WST components with the explanatory variables of teachers’ beliefs towards digital technologies (will), teacher data literacy (skill), and access to data technologies (tool) (Model 1). Lastly, we included additional variables related to the frequency of using digital devices in lessons by teachers and students, and teachers’ characteristics (Model 2). An ANOVA was used to investigate whether the data were better fitted in each model. With respect to the analysis of the different platforms used in schools, we used a t-test to compare teacher data use and data literacy based on whether they had access to different types of digital platforms. All analyses were carried out using RStudio, particularly R 4.1.2. and the packages “pkgs” and “lmerTest.”

## Results

### Descriptive analysis

The three items regarding teacher data use (see Table 1) showed that almost 56% of teachers had access to technologies that provide digital student data but 44.6% of teachers indicated having limited data literacy and 46.7% of teachers disagreed regarding the use of digital student data to inform their teaching practice.

**Table 1:** Proportion of teachers and their responses to the three items of teachers’ data use (weighted percentages)

Teacher data use items	1. Strongly disagree	*2.*	*3.*	*4.*	*5. Strongly agree*
	N (%)	N (%)	N (%)	N (%)	N (%)
*Availability of data technologies*: I have the adequate technology to examine digital student data	73 (6.9)	157 (14.8)	240 (22.7)	323 (30.5)	266 (25.1)
*Teachers’ data literacy*: I know how to improve my teaching and student learning by analyzing digital student data	215 (20.3)	258 (24.3)	311 (29.4)	188 (17.8)	87 (8.2)
*Digital data use by teachers*: I use digital student data to plan and adjust my teaching	236 (22.3)	258 (24.4)	221 (20.9)	162 (15.3)	181 (17.1)

Overall descriptive statistics with regard to the items of the teachers’ questionnaire are reported in Table 2. Descriptive results show that, on average, teachers indicated having medium levels of data literacy and data use. However, the teachers seemed to have access to data technologies, and their beliefs about digital technologies were positive. The use of digital devices by teachers and students was frequent in their lessons.

**Table 2:** Descriptive statistics on teachers’ questionnaire items (weighted data)

Variables	M (SD)
	N = 1059
Digital data use by teachers	2.80 (1.39)
Teachers’ positive beliefs (will)	3.27 (0.92)
Teachers’ data literacy (skill)	2.69 (1.2)
Availability of data technologies (tool)	3.52 (1.2)
Digital device use by teachers	5.59 (0.91)
Digital device use by students	4.62 (1.39)

Section 4.2 presents results on the factors influencing teacher use of digital data that might better explain the above patterns.

### Predictors of teacher digital data use: a multilevel model

The null model estimated whether there were differences in teachers’ digital data use between the schools. The results (see Table 3) showed that between-schools variance differed significantly from zero (σ^2^ = 1.79, χ² = 111.7, p < 0.001) and 10% of the variance in teachers’ digital data use was attributed to differences between schools. We also estimated a model (Model 1) that considered the other three variables related to digital student data and the WST model (positive beliefs-will, teachers’ data literacy-skill, availability of data technologies-tool) as explanatory variables. The results indicated that teachers’ positive beliefs about digital technologies (B = 0.18, p < 0.001), teachers’ data literacy (B = 0.43, p < 0.001) and their access to data technologies (B = 0.32, p < 0.001) were related to teachers’ digital data use for teaching. This suggests that the more teachers perceive themselves as competent in using digital student data for teaching, the more they use this data to inform their teaching. Accordingly, the more teachers have access to data technologies, the more they use digital student data to inform their teaching. Finally, the more positive beliefs teachers have towards digital technologies, the more they use digital data.

In the second model (Model 2), we added teachers’ characteristics (gender, age, teaching years) and the frequency of using digital devices in lessons by teachers and students as predictor variables. The results indicated that the frequency of using digital devices in lessons by students (B = 0.10, p < 0.001) affected teachers’ digital data use for teaching but without any influence of digital device use by teachers (B = 0.00, p = 0.976). Regarding teachers’ characteristics, although gender (B = 0.18, p = 0.003) affected teachers’ digital data use, their age (B = 0.01, p = 0.012) and teaching years (B = -0.01, p = 0.020) slightly affected teachers’ digital data use. The results of the different models are presented in Table 3.

**Table 3:** Model estimates for the two-level analysis of teachers’ digital data use (N = 1043)

	Null model			Model 1			Model 2		
*Predictors*	*Estimates*	*CI*	*p*	*Estimates*	*CI*	*p*	*Estimates*	*CI*	*p*
(Intercept)	2.85	2.70–3.00	**< 0.001**	2.87	2.72–3.02	**< 0.001**	2.87	2.52–3.22	**< 0.001**
Teachers’ positive beliefs (will)				0.18	0.11–0.25	**< 0.001**	0.16	0.09–0.23	**< 0.001**
Teachers’ data literacy (skill)				0.43	0.37–0.49	**< 0.001**	0.43	0.37–0.50	**< 0.001**
Availability of data technologies (tool)				0.32	0.26–0.38	**< 0.001**	0.31	0.25–0.37	**< 0.001**
Digital device use by students							0.10	0.04–0.15	**< 0.001**
Digital device use by teachers							0.00	-0.17–0.54	0.976
Gender							0.18	0.06–0.30	**0.003**
Age							0.01	0.00–0.02	**0.012**
Teaching years							-0.01	-0.02–0.00	**0.020**
**Random Effects**									
σ^2^	1.79			1.02			0.99		
τ_00_	0.19 _School_ID_			0.24 _School_ID_			0.25 _School_ID_		
ICC	0.10			0.19			0.20		
N	54 _School_ID_			54 _School_ID_			54 _School_ID_		
**Model Fit**									
Deviance (2-log)	3620.80			3063.8			3036.4		
χ²	111.7			557			584.93		
Df	52			3			8		
P	< 0.001			< 0.001			< 0.001		
Reference	Single level model			Null model			Null model		
Observations	1043			1043			1043		
Marginal R^2^/Conditional R^2^	0.000/0.097			0.367/0.489			0.380/0.503		

### Platforms used by teachers in different schools

Τhe results with regard to the different digital platforms used by teachers and their schools might provide more information on whether the use of different digital platforms influences the actual use of digital data for teaching and data literacy (see Table 4).

**Table 4:** Number of teachers (N) and descriptive statistics (M, SD) about teachers’ data literacy and use according to teachers’ access to digital platforms in their schools

Digital platforms		Digital data use by teachers		Teachers’ data literacy	
		*Access*	*No access*	*Access*	*No access*
*Reading-Writing*	N	658	385	658	385
	M(SD)	2.74(1.41)	2.86(1.38)	2.61(1.24)	2.73(1.22)
*Learning Management System (LMS)*	N	605	438	605	438
	M(SD)	2.76(1.4)	2.82(1.4)	2.6(1.25)	2.73(1.21)
*App for learning*	N	200	843	200	843
	M(SD)	2.72(1.3)	2.80(1.42)	2.7(1.2)	2.64(1.24)
*Videoconference*	N	274	769	274	769
	M(SD)	2.57(1.36)	2.87(1.4)	2.54(1.22)	2.69(1.23)
*Cloud-based collaboration*	N	500	543	500	543
	M(SD)	2.96(1.38)	2.63(1.4)	2.75(1.23)	2.56(1.23)
*Videoconference/LMS/Collaboration*	N	636	407	636	407
	M(SD)	2.78(1.38)	2.79(1.42)	2.63(1.25)	2.70(1.2)
*Other*	N	286	757	286	757
	M(SD)	2.72(1.42)	2.81(1.39)	2.61(1.25)	2.67(1.22)
**Analytics**					
*Digital platform with analytics*	N	879		879	
	M(SD)	2.78(1.39)		2.65(1.25)	
*Digital platforms without analytics*	N	331		331	
	M(SD)	2.85(1.43)		2.67(1.16)	
*> 2 Platforms with analytics*	N	164		164	
	M(SD)	2.80(1.34)		2.64(1.24)	

Table 4 also shows that 84% of teachers had access to digital platform with analytics and only 16% of teachers did not have access to platforms with analytics. This result further confirms that most teachers had available technologies to examine digital student data in their schools. However, although teachers had access to platforms with analytics, their self-reported use of digital data and data literacy were low. The most commonly used were cloud-based platforms for digital collaboration, platforms for reading/writing, LMSs, and videoconference systems. There was a significant difference in digital data use, t(1041) = 1036, p < 0.001, d = 0.24. and teachers’ data literacy, t (1041) = 1033, p = 0.014, d = 0.15 between teachers who had access to cloud-based platforms for collaboration and those without access to platforms for collaboration in their schools. Access to the other types of platforms shown in Table 4 did not show any significant differences in teachers’ data use and data literacy.

## Discussion

Teachers’ digital data use is an emerging topic due to the increasing use of digital technologies in schools. Although there is initial evidence showing that school teachers lack data literacy skills with regard to data use for informing their teaching, there is a limited number of studies that evaluate this at scale in K–12 education. In addition, few studies have explored relevant factors and types of digital platforms that are associated with more intensive use of digital student data for teaching purposes. Based on our theoretical grounding, models that explain factors of technology use by teachers have been scarcely applied in research on learning analytics in schools. For this reason, we conducted a survey study with N = 1059 school teachers in Switzerland based on the WST model that focuses on teachers’ beliefs, skills and availability of tools in schools. With respect to our first research question, our survey study shows that more than 50% of teachers in gymnasiums, technical, and vocational schools indicated having access to technologies that provide digital student data. Thus, there is an opportunity to use digital student data to better inform their teaching. However, only 30% of teachers reported using digital student data for their teaching and 26% of teachers felt confident in using digital student data for this purpose. These results align with previous studies showing that teachers’ data use for instruction varies across districts or schools (Cho & Wayman, 2014). These studies in schools highlighted the importance of accessing relevant computer data systems to use data in practice (Cho & Wayman, 2014; Schildkamp, 2019), however there is a lack of evidence with respect to digital data and learning analytics use by teachers.

In our second research question, we investigated whether teacher and contextual factors such as the different schools influence teachers’ use of digital data. We employed the WST model of technology integration to systematically investigate these factors that positively predict the pedagogical use of digital data by teachers considering that teachers are nested in schools (Rangel et al., 2016). Teacher data use was operationalized based on related items from research on school contexts (Wayman et al., 2016) and was adopted for the purposes of digital data. Our multilevel model initially showed a variance in teachers’ digital use between schools. This result might be explained by the type of schools participated in the study (e.g., gymnasiums, technical, and vocational schools) and possible different subject teachers that use digital data. Our theoretical assumption that the WST model will be relevant in the context of digital data use in schools is confirmed. In particular, the model showed that teachers’ positive beliefs about digital learning technologies (will), their data literacy (skill), and access to relevant data technologies (tool) positively affected teachers’ digital data use to inform instruction. In particular, teacher data literacy was accessed as the central predictor in our model in upper secondary schools and this aligns with previous research on teacher data literacy in schools (Schildkamp, 2019). The additional factors added to the model showed that the frequency of using digital devices by students in teachers’ lessons affected teachers’ digital data use to inform instruction. However, the frequency of using digital devices by teachers did not affect digital data use because in our sample most teachers responded using a digital device in almost every lesson. Teachers’ characteristics, such as their teaching years and age, slightly influenced data use, but their gender seemed to be an important factor in digital data use.

Our results confirm related studies that have investigated factors such as access to data management systems, teachers’ learning on how to use data, and teachers’ beliefs (Rangel et al., 2016; Datnow & Hubbard, 2016). Our study added that research on teachers’ use of digital data can be guided by the research on technology integration with the WST model. Moreover, relevant studies have found that teachers’ sense-making of educational data is shaped by the context, district, networks, and school leadership (Rangel et al., 2016), and our study shows that differences in teachers’ data use are attributed to the differences in the context of schools. Datnow and Hubbard (2016) offered a model that shows that teachers’ attitudes and beliefs are important in actual data use. When teachers are provided with opportunities to discuss data-related practices with colleagues, they perceive higher control. Howard et al. (2022) highlighted a growing interest in using digital data in schools, which aligns with the adoption of digital technologies and learning management systems (Baker et al., 2020).

In our third research question we focused on the availability of digital platforms available in schools. Our analysis of the different platforms within our sample shows that schools often use cloud-based platforms for collaboration, reading/writing, learning management systems, videoconferencing, and platforms that provide learning analytics. The results show significant differences in teachers’ use of digital data and data literacy when teachers had access to cloud-based platforms for collaboration compared to when they lacked access to these platforms, suggesting that collaborative platforms could provide more opportunities for the pedagogical use of digital data in schools compared to the other platforms’ types included in our analysis. Surprisingly, access to LMSs did not show any differences in the use of digital data. Although the surveyed teachers seemed to have good access to different technologies that provide learning analytics, they reported low levels of data use and data literacy. This could potentially prompt the question of whether the teachers were aware of the data analytics functionalities of the different types of platforms that were at their disposal in their schools. Further studies may evaluate platforms based on the specific learning analytics or personalized learning functions provided to teachers and school administrators to better understand the relationship between access to data technologies, data literacy, and use.

Based on our results, there seems to be a need for better capacity building in teachers’ use of digital data (Farley-Ripple & Buttram, 2015), and this is also relevant to digital platforms that provide digital student data. Capacity building might facilitate teachers’ understanding of the different types of digital data (e.g., assessment, attendance, and student progress) as well as the possible different uses to inform their teaching in schools (Howard et al., 2022). Jimerson & Wayman (2015) argued that “Training for data use often is synchronous with technology training” (p. 36), and therefore, a connection with training on digital technologies for teaching is needed. Finally, specific plans for the development of data-informed practices in schools, as recommended in previous research (Jimerson & Wayman, 2015), remain pertinent. Our results add more evidence to large-scale studies on teacher data use (Keuning et al., 2019; Kippers et al., 2018; Pierce et al., 2013; Prenger & Schildkamp, 2018; Yan et al., 2021) and expand the findings in the case of digital student data or learning analytics in the K–12 context (Hase et al., 2022; Mavroudi et al., 2021) showing that different school contexts and digital platform types can influence teachers’ use of learning analytics (Sousa et al., 2021).

## Conclusions

Our study shows that teachers differ in the use of digital data, and this is influenced by teachers’ positive beliefs towards digital technologies (will), the self-perceived competency in using these platforms (skill), the available technologies (tool) and the general frequency of using digital devices in lessons by students. Thus, we were able to confirm and expand the application of the WST to teachers` data use. Our results confirm previous research on teacher data literacy in schools and provide further evidence regarding the digital data literacy of teachers. This is also one of the first studies that provide empirical evidence with regard to teacher data literacy and learning analytics in school contexts. Our study has certain limitations, including the fact that we measured teachers’ beliefs regarding digital technologies in general instead of digital data in particular. In addition, due to the constraints of survey length, we were only able to use single items to measure teacher data use, access to technologies, and teacher data literacy. Further studies might construct scales related to teacher data use with items relevant to teacher data literacy. In addition, we did not restrict our evaluation to specific types of digital data (e.g., assessment data and participation data); instead, we relied on teacher responses regarding various types of digital data provided on digital platforms. Future studies might focus on a specific type or different category of digital data that might inform teaching. However, our main purpose was to understand teachers’ perceptions and factors of digital data use on a larger scale in schools. More detailed research is needed regarding various factors, such as the school context, access to available technologies, the frequency of using digital devices, teachers’ positive beliefs toward digital technologies, and teacher competency with digital data. Finally, teacher training in the use of digital data provided in learning analytics tools is recommended to develop teacher data literacy in schools.

## Data Availability

The data that has been used is confidential.
